# Establishment of patient-derived xenografts from patients with gastrointestinal stromal tumors: analysis of clinicopathological characteristics related to engraftment success

**DOI:** 10.1038/s41598-020-64552-w

**Published:** 2020-05-14

**Authors:** Young-Soon Na, Min-Hee Ryu, Young Soo Park, Chae-Won Lee, Ju-Kyung Lee, Yangsoon Park, Jung Min Park, Jungeun Ma, Yoon-Koo Kang

**Affiliations:** 1 Asan Institute for Life Sciences, Asan Medical Center, University of Ulsan College of Medicine, Seoul, Korea; 2 Department of Oncology, Asan Medical Center, University of Ulsan College of Medicine, Seoul, Korea; 3 Department of Pathology, Asan Medical Center, University of Ulsan College of Medicine, Seoul, Korea

**Keywords:** Cancer models, Gastrointestinal cancer, Cancer models

## Abstract

Patient-derived xenografts (PDXs) can represent the heterogeneity and histological characteristics of tumors and are thus useful for testing the efficacy of anti-cancer drugs; however, PDXs are difficult to generate, especially for gastrointestinal stromal tumor (GIST). We analyzed the clinicopathologic factors associated with the successful establishment of GIST PDX in NOD.Cg-*Prkdc*^*scid*^ *IL2rg*^*tm1Wjl*^/SzJ mice. We used 185 GIST tumor fragments from patients who underwent surgical resection prior to (n = 66; 35.7%) and after treatment (n = 119; 64.3%) with tyrosine kinase inhibitors. The overall success rate of PDX establishment was 17%; in univariate analysis, engraftment success was associated with after TKI treatment, larger tumor size, higher mitotic count, higher Ki-67 index, higher cellularity, presence of tumor necrosis, primary mutations in *KIT* exon 11, and originating from metastatic lesions. In multivariate analysis, higher Ki-67 index, after TKI treatment, and larger tumor size were independent factors for engraftment success. Immunohistochemistry in representative samples further corroborated the above results. These results will be useful in the establishment of PDX models from GISTs.

## Introduction

In the development of anti-cancer drugs, drug responses are tested on cell lines or xenografts. However, as these models cannot accurately represent the patient’s tumor status, they cannot reflect drug efficacy as well^1,2^. Therefore, it is necessary to develop a system in which the tumor status of a patient is well-reflected to develop efficient and specific anti-cancer agents and to study the biological characteristics of each cancer type^3,4^. For this purpose, patient-derived xenograft (PDX) models are widely used.

Even though PDXs are time-consuming and costly for preclinical research, they are more accurate than cell lines and cell line xenografts in representing tumor heterogeneity, histological characteristics of the original tumor and stromal compartment at the early stage^5^. PDXs also retain many molecular features of the original patient tumors, and the clinical response concordance is better in PDXs than in cell lines and cell line xenografts^2,6^. Therefore, PDX models are helpful in cancer studies^6,7^. PDXs have been established in various tumor types with varying success rates, ranging from 95% in prostate cancer to 9% in renal cell carcinoma^1,2^. For soft tissue sarcomas, the success rates of PDXs have been reported to be 37.8 to 70.9%^8^. However, only a few gastrointestinal stromal tumor (GIST) PDX models have been reported to date.

GIST is the most common mesenchymal tumor of the gastrointestinal tract, and is characterized by mutations in the *KIT* or platelet-derived growth factor receptor alpha (*PDGFRa*) genes. Accordingly, tyrosine kinase inhibitors (TKIs) targeting these active mutations (i.e., imatinib, sunitinib, and regorafenib) have shown promising results and are therefore approved for the treatment of GIST through clinical trials. Unfortunately, most of the patients eventually show disease progression even after receiving these drugs^9,10^.

The initial report on the GIST PDX models with mutations of *KIT* exon 11 was published by Huynh *et al*.^11^. UZLX-GIST9 harboring both primary and secondary mutations in *KIT* exons 11 and 17 was established from a metastatic lesion that showed clinical progression after treatment with imatinib, sunitinib, and regorafenib. GIST PDX models harboring primary mutation alone in either *KIT* exon 9 or exon 11 were also reported^12–16^. We have previously reported the establishment of 3 separate GIST PDX models - GIST-RX1 (mutations in *KIT* exons 11, 17, and *PTEN* from a patient with resistance to imatinib, sunitinib, and sorafenib), GIST-RX2 (mutations in *KIT* exons 11 and 14 from a patient with resistance to imatinib), and GIST-RX4 (mutations in *KIT* exons 9 and 17 from a patient with resistance to imatinib and sunitinib)^17^. Some GIST PDXs are commercially available, but they do not have various *KIT* mutations.

Previous studies have shown that the successful establishment of PDXs is critically influenced by factors such as characteristics of tumor tissue or the process of PDX establishment^5,18^. Therefore, we examined the clinicopathological characteristics associated with the successful establishment of GIST PDXs.

## Results

### Clinical characteristics of the GIST patients at tissue collection

The clinical characteristics of the 176 patients with GIST (185 samples) at tissue collection are shown in Table 1. The median age was 59 years, and the primary sites were mostly the stomach (47.0%) and small bowels (47.6%). A total of 66 (35.7%) samples were from treatment-naïve patients, 119 (64.3%) were from patients after TKI treatment at the time of resection. The largest tumor size in the majority of samples was ≤50 mm (n = 93; 50.3%). Primary mutations were mostly located in *KIT* exon 11 (62.7%) and exon 9 (14.1%), with a minor portion of samples harboring primary mutations in *KIT* exon 17 or *PDGFRA* exon 18. Approximately one-third (35.7%) of the samples were from patients who had localized resectable disease and had not received TKI therapy at the time of tissue collection, while the other two-thirds (64.3%) were from patients who had received TKI therapy. The median treatment durations with imatinib, sunitinib, and regorafenib were 34.4, 11.9, and 11.1 months, respectively.

**Table 1:** Clinical characteristics of the GIST patients at tissue collection.

Characteristics	No. (%)
**Age (year)**	
median (range)	59 (28–88)
≤59	93 (50.3)
>59	92 (49.7)
**Sex**	
male	97 (52.4)
female	88 (47.6)
**Primary site**	
stomach	87 (47.0)
small bowel	88 (47.6)
large intestine	8 (4.3)
others	2 (1.1)
**Disease status**	
before TKI	66 (35.7)
after TKI	119 (64.3)
**Largest tumor size (mm)**	
≤ 50	93 (50.3)
50–100	60 (32.4)
>100	32 (17.3)
^a^**Primary mutation**	
*KIT* exon 9	26 (14.1)
*KIT* exon 11	116 (62.7)
Others	12 (6.5)
Wild type	20 (10.8)
NE	11 (5.9)
**Resection site for PDX**	
primary	87 (47.0)
metastasis	98 (53.0)
**Drug exposure**	
No	66 (35.7)
Imatinib alone	84 (45.4)
Imatinib and Sunitinib	25 (13.5)
Imatinib, Sunitinib, and Regorafenib	10 (5.4)
**Duration of TKI (months)**	
Imatinib, median (range)	34.4 (0.9–145.9)
Sunitinib, median (range)	11.9 (0.7–58.1)
Regorafenib, median (range)	11.1 (3.7–32.9)

TKI: tyrosine kinase inhibitor.

Wild type: non-KIT and non-PDGFR mutant.

PDX: patient-derived xenograft.

NE: not evaluated.

^a^Mutation analysis in *KIT* exons 9, 11, 13, 14, and 17, and *PDGFRa* exons 12 and 18 by Sanger sequencing.

### Clinicopathological characteristics of patients with successful PDX establishment

We successfully established 31 GIST PDX models from 185 samples (16.8%), including the previously reported 3 PDX models^17^. The clinicopathological characteristics of the cases with successful PDX establishment are summarized in Table 2. Four PDX models were established from localized tumor samples and 27 were established from metastatic tumor samples. Thirty PDX models were established from GIST lesions resistant to imatinib, sunitinib, and/or regorafenib, and only one was established from a GIST patient prior to TKI treatment. The clinicopathological characteristics of the cases with unestablished PDX are summarized in Supplementary Table S1.

**Table 2:** Characteristics of the GIST patients with successful PDX establishment.

PDX	Patient											
	No.	Age	Sex	Primary site	^a^Resection site	Largest tumor size (mm)	Mitotic count (/50 HPFs)	Ki-67	Cellularity	Tumor necrosis	Primary mutation	^b^Drug exposure (months)
GIST-RX1	1	67	F	small bowel	peritoneum (M)	108	500	≥1/3	high	yes	*KIT* exon 11	I (43) S (11) So (2)
GIST-RX2	2	42	M	stomach	stomach (P)	76	51	<1/3	high	no	*KIT* exon 11	I (21.5)
GIST-RX3	3	43	M	small bowel	peritoneum (M)	174	88	≥1/3	high	yes	*KIT* exon 11	I (49.5) S (58.1)
GIST-RX4	4	79	M	small bowel	liver (M)	250	129	≥1/3	high	yes	*KIT*exon 9	I (30.3) S (5.6)
GIST-RX5	5	40	M	small bowel	small bowel (P)	57	45	<1/3	high	yes	*KIT* exon 11	I (95.6)
GIST-RX6	5	40	M	small bowel	peritoneum (M)	131	>150	≥1/3	high	no	*KIT* exon 11	I (101.4) S (0.9)
GIST-RX7	6	42	M	others	peritoneum (M)	67	300	≥1/3	high	yes	*KIT* exon 11	I (105.3) S (0.7)
GIST-RX8	7	61	M	small bowel	peritoneum (M)	54	119	≥1/3	high	yes	*KIT* exon 11	I (88.1) S (4.6)
GIST-RX9	8	49	F	small bowel	liver (M)	136	140	≥1/3	high	yes	*KIT* exon 11	I (41.6) S (21) R (6)
GIST-RX10	9	60	F	stomach	peritoneum (M)	90	110	≥1/3	high	yes	*KIT* exon 11	I (0.3) S (22.8)
GIST-RX11	10	50	M	small bowel	liver (M)	230	20	<1/3	low	yes	*KIT* exon 11	I (98.6) S (11.6)
GIST-RX12	11	76	F	stomach	peritoneum (M)	215	60	≥1/3	high	yes	*KIT* exon 11	I (19.4)
GIST-RX13	12	72	F	small bowel	peritoneum (M)	73	115	≥1/3	high	yes	WT	I (11.3)
GIST-RX14	13	54	F	small bowel	liver (M)	50	5	≥1/3	low	no	*KIT* exon 11	I (34.2) S (22.3)
GIST-RX15	14	55	F	stomach	liver (M)	81	138	≥1/3	high	yes	*KIT* exon 11	I (33)
GIST-RX16	15	32	F	stomach	liver (M)	110	22	≥1/3	high	yes	*KIT* exon 11	I (10.8)
GIST-RX17	16	50	M	small bowel	peritoneum (M)	29	46	≥1/3	high	no	*KIT* exon 11	I (34.6)
GIST-RX18	17	57	F	stomach	liver (M)	148	125	≥1/3	high	yes	*KIT* exon 11	I (145.9) S (5.1)
GIST-RX19	18	67	M	stomach	peritoneum (M)	59	50	<1/3	low	yes	*KIT* exon 11	I (40.5) S (18.7)
GIST-RX20	19	64	M	stomach	peritoneum (M)	131	74	≥1/3	high	yes	*KIT* exon 11	I (29) S (16.3) R (32.9)
GIST-RX21	20	55	M	others	liver (M)	163	51	≥1/3	high	yes	^c^*KIT* exon 11, *PDGFR* exon 18	I (58.8), S (18.1) R (32.9)
GIST-RX22	21	49	M	small bowel	liver (M)	64	65	≥1/3	high	yes	WT	I (31.5) S (4.1)
GIST-RX23	22	63	F	stomach	peritoneum (M)	26	26	<1/3	low	no	*KIT* exon 11	I (72.2)
GIST-X24	23	77	F	stomach	stomach (P)	150	37	≥1/3	high	yes	*KIT* exon 11	None
GIST-RX25	24	50	M	stomach	stomach (P)	45	37	≥1/3	high	yes	*KIT* exon 11	I (5.8)
GIST-RX26	25	69	M	stomach	liver (M)	76	123	≥1/3	high	no	*KIT* exon 11	I (19.5) S (14.3)
GIST-RX27	26	66	F	others	peritoneum (M)	62	16	<1/3	high	no	*KIT* exon 9	I (14.5)
GIST-RX28	27	70	M	stomach	peritoneum (M)	20	320	≥1/3	high	yes	*KIT* exon 11	I (24.6)
GIST-RX29	16	52	M	small bowel	peritoneum (M)	80	97	≥1/3	high	yes	*KIT* exon 11	I (56) S (3.9) R (3.7)
GIST-RX30	28	71	M	small bowel	liver (M)	27	40	<1/3	high	no	*KIT* exon 9	I (91)
GIST-RX31	29	81	M	small bowel	peritoneum (M)	82	36	≥1/3	high	yes	*KIT* exon 11	I (22.1)

^a^M, metastasis; P, primary tumor.

^b^I, imatinib; S, sunitinib; R, regorafenib; So, sorafenib.

^c^*KIT* exon 11, *PDGFR* exon 18: The mutation analysis of *PDGFR* was not performed at the time of diagnosis, so it was not possible to confirm whether it is a double mutation of *KIT* exon 11 and *PDGFR* exon 18. Mutations in *KIT* exon 11 and *PDGFR* exon 18 were both found at the time of resistance to imatinib and at the time of PDX establishment.

WT, wild type.

There were two cases from whom distinct PDXs had been established at different timepoints. GIST-RX5 and GIST-RX6 were established from a patient at the time of progressive disease while receiving imatinib and sunitinib, respectively. GIST-RX17 and GIST-RX29 were established from a patient at the time of progressive disease while receiving imatinib and regorafenib, respectively. In one patient, PDX was not established when the sample was obtained during progressive disease after 800 mg imatinib, but a later sample obtained after re-challenge with imatinib was successfully established as a PDX (GIST-RX23). In another patient, a sample obtained during sunitinib treatment was successfully established as a PDX (GIST-RX8), and a later sample obtained after re-challenge with imatinib after progression is being monitored for tumor formation in F1. Nine samples from four patients were not established as PDX even when the samples were obtained after different responses to drugs.

### Clinicopathological characteristics related to engraftment success

We examined the clinicopathological characteristics associated with the successful establishment of PDX (Table 3). In univariate analysis, factors such as age, sex, primary tumor sites, and cell types did not show a significant association with PDX engraftment success rate. Conversely, the majority of successful samples (96.8%, 30/31) were from patients after TKI treatment (*p* = 0.003). In terms of largest tumor size, the success rate was the highest in the >100 mm group (37.5%) and lowest in the ≤ 50 mm group (6.5%, *p* < 0.001). As for mitotic count, 58 (31.4%) and 127 (68.6%) samples had mitotic counts of ≤ 5/50 high power fields (HPFs) and >5/50 HPFs; importantly, the >5/50 HPF group had a significantly higher proportion of successful engraftment (23.6% vs. 1.7%, *p* < 0.001). The success rate also significantly differed between samples with necrosis and those without (26.4% vs. 8.3%, *p* = 0.001). Samples with Ki-67 expression of ≥1/3 had a significantly higher success rate than those <1/3 (42.9% vs. 5.4%, *p* < 0.001). Samples with high cellularity had a higher success rate (26.2%) than did those with low cellularity (4.9%, *p* < 0.001). Notably, the PDX engraftment success rate was higher in samples with primary mutations in *KIT* exon 11 (22.4%) than in others (8.6%, *p* = 0.031). Lastly, samples from metastatic sites had a higher success rate than those from primary sites (27.6% vs. 4.6%, *p* < 0.001).

**Table 3:** Clinicopathological characteristics related to successful engraftment of GIST PDX.

Variables	Success (N = 31)		Failure (N = 164)		Univariate	Multivariate	
	N	%	N	%	*p*-value	OR (95% CI)	*p*-value
Age					0.870		
>59	15	16.3	77	83.7			
≤59	16	17.2	77	82.8			
Sex					0.279		
female	12	13.6	76	86.4			
male	19	19.6	78	80.4			
Primary site					0.534		
stomach	13	14.9	74	85.1			
others	18	18.4	80	81.6			
Disease status					0.003		0.035
before TKI	1	1.5	65	98.5		1	
after TKI	30	25.2	89	74.8		9.437 (1.730-176.100)	
Largest tumor size (mm)					<0.001		0.057
≤50	6	6.5	87	93.5		1	
50-100	13	21.7	47	78.3		2.855 (0.941-9.464)	0.071
>100	12	37.5	20	62.5		4.197 (1.285-14.835)	0.020
Mitotic count (/50 HPFs)					<0.001		
≤5	1	1.7	57	98.3			
>5	30	23.6	97	76.4			
Ki-67					<0.001		<0.001
<1/3	7	5.4	122	94.6		1	
≥1/3	24	42.9	32	57.1		7.317 (2.880-20.582)	
Cellularity					<0.001		
low	4	4.9	78	95.1			
high	27	26.2	76	73.8			
Tumor necrosis					0.001		
absent	8	8.3	88	91.7			
present	23	26.4	64	73.6			
NE	0		2				
Cell type					0.520		
spindle	18	14.8	104	85.2			
epithelioid	4	19.0	17	81.0			
mixed	9	22.5	31	77.5			
NE	0		2				
Primary mutation					0.031		
others	5	8.6	53	91.4			
*KIT* exon 11	26	22.4	90	77.6			
NE	0		11				
Resection site for PDX					<0.001		
primary	4	4.6	83	95.4			
metastasis	27	27.6	71	72.4			

NE, not evaluated.

Significant factors (p < 0.05) from the univariate analysis were included in the multivariate analysis.

Variable selections were performed by backward elimination.

We assessed the multicollinearity of the final model by using variance inflation factors. Univariate analysis showed that factors such as after TKI treatment, largest tumor size, mitotic count, Ki-67, cellularity, necrosis, primary mutation, and tumors originating from metastatic lesions were significantly associated with the successful establishment of PDX. Among them, the following remained as significant factors for success in multivariable analysis with backward elimination: Ki-67 index of ≥1/3 (odds ratio [OR]: 7.317, 95% confidence interval [CI]: 2.880–20.582; *p* < 0.001), after TKI treatment (OR: 9.437, 95% CI: 1.730–176.100; *p* = 0.035), and largest tumor size of >100 mm (OR: 4.197, 95% CI: 1.285–14.835; *p* = 0.020) and 50–100 mm (OR: 2.855, 95% CI: 0.941–9.464; *p* = 0.071) (overall *p* = 0.057). We analyzed the success rate according to the number of three factors related to success (i.e., Ki-67 ≥ 1/3, after TKI treatment, and largest tumor size [>50 or >100]) as shown in Supplementary Table S2. The greater the number of factors related to success, the more we can consider these as high-engrafters.

**Figure 1:**
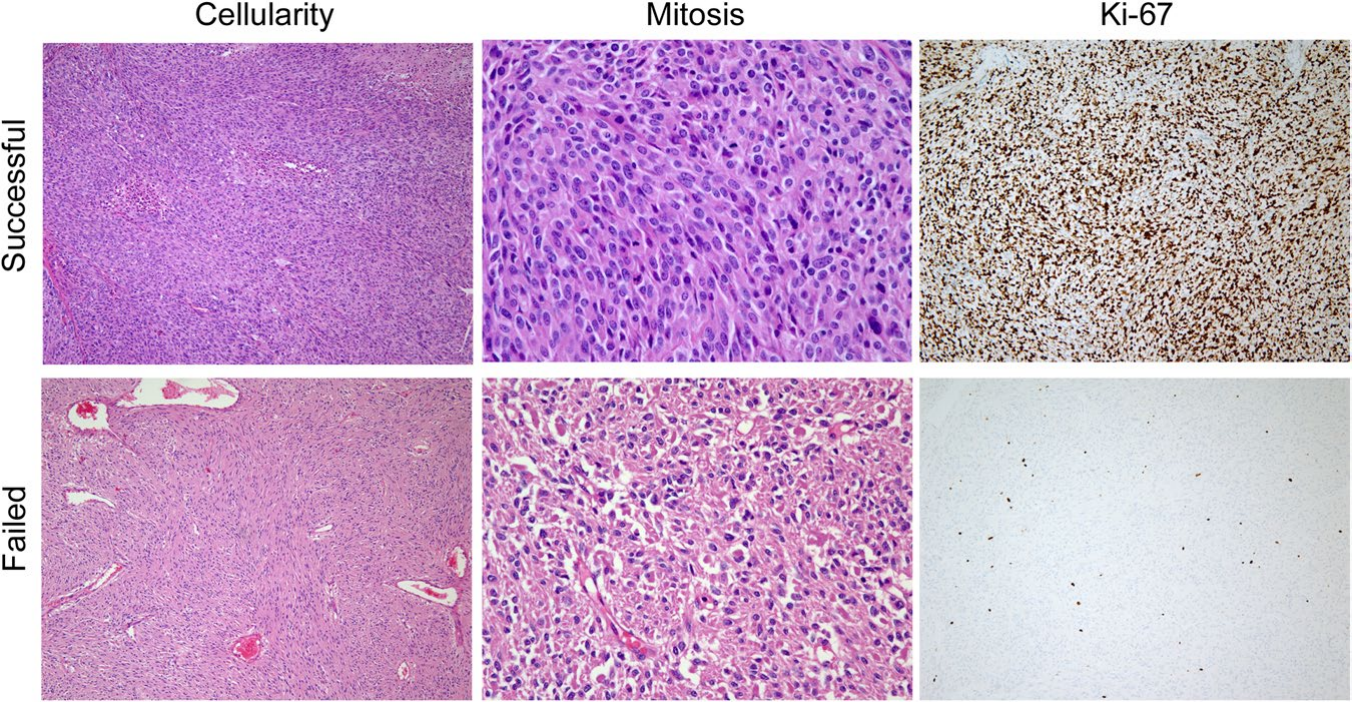
Representative photomicrographs of histomorphology and Ki-67 immunohistochemical staining. Upper row shows a successful case of PDX (RX18) and lower row shows a failed case. The first column denotes higher cellularity in a successful case (original magnification x100). In the second column, frequent mitotic figures are observed in the successful case but none in the failed case (original magnification, x400). The third column contrasts high versus low Ki-67 staining.

We performed immunohistochemistry on all samples. Figure 1 shows the representative images of a successful sample (GIST-RX18) and an unsuccessful sample for PDX establishment. Compared with unsuccessful samples, successful samples had higher cellularity, higher mitotic count, and higher Ki-67 expression, indicating that these factors are indeed related to successful PDX establishment.

## Discussion

We analyzed 12 clinicopathologic factors to determine the factors associated with successful PDX engraftment. Our multivariate analysis showed that the Ki-67 index was an independent factor associated with PDX engraftment success; also, after TKI treatment and the largest tumor size showed some association with PDX success as well. As previously reported, short tandem repeat (STR) analysis, histological analysis, mutation analysis, and monitoring response to TKI were performed in patients and their matched established tumors from the PDX models to validate the established PDXs^17^. Among the patients with GIST who develop resistance to TKIs, some cases lose KIT expression over time for yet unknown reasons. However, in our PDX models, KIT expressions were confirmed using immunohistochemistry in all patients at the time of establishment and remained evident in Western blotting in the 4^th^-5^th^ passage (F4-F5) PDXs that have been investigated to date. We are currently conducting a molecular-level analysis of the established 28 models, three of which have been characterized in our previous report^17^; we are also monitoring the 28 models in terms of their responses to TKI^17^. The response of the PDX models to TKIs-specifically, the results of the imatinib responses-were consistent with the clinical resistance, although there were some differences in the response to TKIs among the TKI-resistant PDX models. We believe that our GIST PDX models may be useful for assessing the efficacy of new drugs in TKI-resistant GIST and for investigating the molecular and cellular mechanism of TKI resistance.

The success rate of PDX is affected by multiple factors. Shorter devascularized time from tumor resection to implantation in mice was shown as the main determinant for successful PDX engraftment^18^. However, another study reported that time to engraftment was not significantly related to successful engraftment^19^. Maintenance of tissues in cold and fresh media immediately after surgery, the size and number of implanted tissue, and the location of implantation may affect the success rate^2^. Stroma and endothelial cells, regulators of tumor growth, are also important^20,21^. Among the immunodeficient mouse strains used for PDX, the NSG mice are more suitable than other strains such as nude, severely compromised immune deficient (SCID), and nonobese diabetic (NOD)-SCID (NOD.CB17/*Prkdc*^*scid*^/J)^22^. The growth rates of patient tumors are highly variable^23^; similarly, the PDX models in our study exhibited varying growth rates with a median of 4.6 months (range, 1.5–11) for reaching 2 cm in diameter after implantation.

The aggressiveness and histological type of tumor as well as tumor cell proportion were considered important for successful PDX establishment^2^. Mitotic count, Ki-67, and cellularity are related to tumor cell proliferation and mitotic count itself is correlated closely with Ki-67 and cellularity^24^. High degrees of mitotic index, Ki-67 expression, and tumor cellularity were risk factors for the aggressive biological behavior of GISTs^25,26^. Therefore, factors related to malignant potentials seem to be associated with engraftment success. The presence of tumor necrosis was also correlated with a high risk for malignancy^27^. Patients who develop acquired resistance after TKI treatment are associated with aggressive clinical behavior^28^. As tumor size is associated with recurrence and resistance to TKIs in GIST^29,30^, a large-sized GIST can be related to rapid tumor progression and affect PDX engraftment success rate. A previous study has shown that metastatic tumors are more effective than primary tumors in yielding PDX^19^. Therefore, our results suggest that clinicopathologic factors associated with high risk for malignancy may influence the success of PDX engraftment.

*KIT* mutations are found in 60–85% of GISTs, while *PDGFRA* exon 12, 14, and 18 mutations are found in 5–10%^31^. Primary *KIT* mutations in GIST are found mostly in exon 11 (61–71%), less in exon 9 (7–15%), rarely in exons 17 (0.5–1%) and 13 (0.5–1.8%), and extremely rare in exon 8 (0.15–0.23%)^28^. Resistance to TKIs and tumor progression are known to be influenced by genotypes. Generally, patients with primary *KIT* exon 11 mutant GISTs show better treatment outcomes with imatinib and regorafenib, in contrast to poor treatment outcomes with sunitinib^28,32–34^. Since our samples are all mixed up with tissues after imatinib, sunitinib, or regorafenib, the difference in success rate by genotype may not be statistically significant. *KIT* exon 11 mutations containing p.W557_K558 deletion were classified as high risk^28^. However, in our current study, these mutations were not significantly related to the PDX success rate. To date, there are no reports on GIST cell lines that harbor mutations in *KIT* exon 9. As for GIST PDX models, the GIST-RX4 model in our previous report^17^, UZLX-GIST2^15^, and one commercially available model have *KIT* exon 9 mutations. In addition, two additional PDX models (GIST-RX27 and GIST-RX30) established in this study harbor mutations in *KIT* exon 9. As mutations in *KIT* exon 9 are rarer and more resistant to imatinib compared with mutations in *KIT* exon 11, its establishment as a PDX model holds critical research values.

In conclusion, we found that clinicopathologic factors such as after TKI treatment, large tumor size, high mitotic count, high Ki-67 index, high cellularity, presence of tumor necrosis, primary mutation in *KIT* exon11, and metastatic tumor lesions were associated with a higher success rate of PDX establishment. Especially, Ki-67 index, after TKI treatment, and largest tumor size were notable factors for successful PDX engraftment. These findings may be helpful in assisting the establishment of PDX models from GISTs. Yet, additional studies are needed to improve the establishment of difficult-to-engraft GISTs such as those with deficiencies in succinate dehydrogenase.

## Materials and Methods

### Establishment of GIST PDX models

To establish patient-derived GIST xenografts, we implanted GIST tumor fragments from 185 samples of 176 Korean patients who underwent surgical resection prior to and after treatment with tyrosine kinase inhibitors from July 2012 to July 2017 in NOD.Cg-*Prkdc*^*scid*^ *IL2rg*^*tm1Wjl*^/SzJ (NSG) mice (Jackson Laboratory, Bar Harbor, ME, USA). Tumor and peripheral blood samples were collected from patients who provided written informed consent. This study was approved by the Institutional Review Board and the Institutional Animal Care and Use Committee of Asan Medical Center (Seoul, Korea, IRB No. 2017–13–266). All methods in this study were performed in accordance with the relevant guidelines and regulations.

Resected GIST lesions were immediately stored in a chilled medium, and the tumors were diced into 2- to 3-mm pieces and subcutaneously transplanted into each hind side of the flank, especially near the axilla in 6- to 10-weeks-old NSG mice^17^. After reaching 1.5–2 cm in long-axis diameter, the tumors were excised, cut into small pieces under sterile condition, and transplanted into successive BALB/c nude mice^17^. The PDXs were initially generated in F0 mice, then implanted in F1 mice. The established PDXs were passaged to generations greater than F2. Pathologic diagnosis of the GISTs in the PDXs was assessed by a qualified pathologist and lymphomas were excluded to determine successful PDX.

### Clinical information

We gathered the clinical information of the GIST patients including age, sex, resection site, disease status, largest tumor size on computed tomography scan, mitotic count, presence or absence of tumor necrosis, Ki-67, cellularity, cell type, primary mutation, and resection site of primary/metastatic tumor for PDX at the time of engraftment of PDX tumors.

### Immunohistochemistry and hematoxylin-eosin staining

Formalin-fixed, paraffin-embedded, 4-µm tumor sections were dewaxed in xylene, rehydrated with graded alcohol concentrations, and placed in an endogenous peroxide blocking buffer for 15 minutes^17^. Sections were washed in water, antigen-retrieved, and placed in citrate buffer^17^. Nonreactive staining was blocked by treating the sections with 1% horse serum in Tris-buffered saline (pH 6.0) for 3 minutes^17^. Ki-67 antibody (clone MIB1, IS626, 1:200; DAKO, Denmark) was then applied and the binding of antibodies was detected using the avidin-biotin-peroxidase complex (Universal Elite ABC Kit; Vectastain, Burlingame, CA, USA) for 10 minutes. Diaminobenzidine tetrahydrochloride solution (Kit HK153–5K; Biogenex, San Ramon, CA, USA) was used as a chromogen^17^. The tumor specimens were stained with hematoxylin and eosin (H&E) for the examination of the basic histomorphological features^17^.

### Statistical analysis

All analyses were performed using SPSS version 23 (IBM Corp., Armonk, NY, USA) and R (version 3.6.1). *P*-values < 0.05 were considered statistically significant. The Chi-squared test was used to compare categorical variables. Logistic regression analysis was performed to verify significant factors for successful PDX establishment. Factors that showed significant results from the univariate analysis were included in the multivariate analysis with backward elimination.

## Supplementary information

Supplementary information.

Supplementary information2.
