# Supplementary material for: Establishment of patient-derived xenografts from patients with gastrointestinal stromal tumors: analysis of clinicopathological characteristics related to engraftment success
Source: Sci Rep. 2020 May 14;10:7996. doi: 10.1038/s41598-020-64552-w (PMC7224375; doi:10.1038/s41598-020-64552-w)
Supplement: Supplementary file 1 — Supplementary information. [file 41598_2020_64552_MOESM1_ESM.docx]

**Establishment of patient-derived xenografts from patients with gastrointestinal stromal tumors: analysis of clinicopathological characteristics related to engraftment success**

Young-Soon Na^1†^, Min-Hee Ryu^2†^, Young Soo Park^3^, Chae-Won Lee^1^, Ju-Kyung Lee^1^, Yangsoon Park^3^, Jung Min Park^1^, Jungeun Ma^2^, and Yoon-Koo Kang^2*^

^1^Asan Institute for Life Sciences, Asan Medical Center, University of Ulsan College of Medicine, Seoul, Korea

^2^Department of Oncology, Asan Medical Center, University of Ulsan College of Medicine, Seoul, Korea

^3^Department of Pathology, Asan Medical Center, University of Ulsan College of Medicine, Seoul, Korea

^†^Young-Soon Na and Min-Hee Ryu contributed equally as first authors to this work.

**Running title**: Clinicopathological factors related to engraftment success

**Keywords:** Gastrointestinal stromal tumor, patient-derived xenograft, clinicopathological characteristics, success factor

***Correspondence to:** Yoon-Koo Kang, MD, PhD, Department of Oncology, Asan Medical Center, University of Ulsan College of Medicine, 88, Olympic-ro 43-gil, Songpa-gu, Seoul 05505, Korea

Tel.: 82-2-3010-3230; Fax: 82-2-3010-8046; E-mail:[ykkang@amc.seoul.kr](mailto:ykkang@amc.seoul.kr)

Supplementary Table 1. Characteristics of the GIST patients with unestablished PDX

| **Unestablished Patient**  **PDX No. Age Sex Primary ^a^Resection Largest Mitotic Ki-67 Cellularity Tumor Primary ^b^Drug**  **site site tumor count necrosis mutation exposure**  **size (mm) (/50 HPFs) (months)** |
| --- |
| **G1**  30 54 F small peritoneum 116 37 <1/3 high no *KIT* I (50.4),  bowel (M) exon 11 S (2.3),  R (6.2)  **G2**  31 53 M stomach stomach 120 57 <1/3 low yes *KIT*  I (24)  (P) exon 11  **G4** 32 60 M small small 81 175 <1/3 high yes *KIT*  I (18.4)  bowel bowel (P) exon 11  **G5** 33 55 F small peritoneum 169 50 <1/3 high yes *KIT*  I (32),  bowel (M) exon 9 S (3.9)  **G6**  34 76 M stomach stomach 124 355 ≥1/3 high yes WT I (10)  (P)  **G7** 35 52 M stomach stomach 65 0 <1/3 low yes *KIT*  I (15.8)  (P) exon 11  **G8** 36 55 M small peritoneum 105 114 <1/3 high yes *KIT* I (64.6)  bowel (M) exon 11 S (14.1)  **G9** 37 61 F small small 33 31 <1/3 low no *KIT*  I (59.9)  bowel bowel (P) exon 11  **G11** 38 54 F small small 71 0 <1/3 low no *KIT*  None  bowel bowel (P) exon 9  **G15** 39 45 F small peritoneum 98 29 ≥1/3 high yes *KIT*  I (13)  bowel (M) exon 11  **G16** 40 55 M stomach stomach 339 44 ≥1/3 high yes *KIT* I (18.4)  (P) exon 11  **G17** 41 61 M stomach stomach 65 0 <1/3 low no *KIT*  I (12.6)  (P) exon 11  **G19** 42 60 M stomach peritoneum 18 23 <1/3 high no *KIT* I (109.7)  (M) exon 11  **G20** 43 56 M stomach stomach 76 7 <1/3 high no *KIT*  None  (P) exon11  **G21** 44 51 M small peritoneum 89 14 <1/3 high yes *KIT*  I (71.5),  bowel (M) exon 11 S (4.6)  **G25** 45 51 M small small 111 2 <1/3 low no *KIT*  None  bowel bowel (P) exon 9  **G27** 46 57 M small peritoneum 170 20 <1/3 high yes *KIT*  I (10.5),  bowel (M) exon 9 S (1.7),  R (6.2)  **G28** 47 64 F small peritoneum 29 3 <1/3 low no *KIT*  I (87.5)  bowel (M) exon 9  **G29** 48 74 F stomach stomach 81 1 <1/3 low no NE I (0.9)  (P)  **G30** 49 74 F stomach stomach 60 14 <1/3 high no WT None  (P)  **G35** 50 57 F stomach liver 40 3 <1/3 low no *KIT* I (69.5)  (M) exon 11  **G39**  51 60 F others rectum 71 1 <1/3 low no *KIT* I (12.1)  (P) exon 11  **G40** 52 70 M stomach stomach 32 9 <1/3 low no *KIT*  None  (P) exon 11  **G41** 53 36 M stomach stomach 95 130 <1/3 high no WT I (6.4)  (P)  **G42** 54 48 F stomach stomach 38 12 <1/3 low no WT None  (P)  **G45** 55 59 F stomach liver 90 127 ≥1/3 high yes *KIT* I (64),  (M) exon 11 S (14),  R (7.9)  **G46** 56 74 M stomach stomach 52 1 <1/3 low yes *PDGFR* None  (P) exon 18  **G47** 57 62 M small peritoneum 23 13 <1/3 low yes *KIT* I (70.8)  bowel (M) exon 11  **G48** 58 48 F small bone 31 66 ≥1/3 low no *KIT*  I (62.3)  bowel (M) exon 11 S (4.2)  R (13.1)  **G49** 59 66 M stomach stomach 77 6 <1/3 low no *PDGFR*  None  (P) exon 18  **G52**  60 39 M small peritoneum 55 40 <1/3 high no WT I (36.8),  bowel (M) S (19.6)  **G54** 61 75 M stomach stomach 87 5 <1/3 low yes *KIT*  None  (P) exon 11  **G55** 62 58 F small peritoneum 51 9 <1/3 high no *KIT*  I (84.9)  bowel (M) exon 11  **G56** 63 65 M small peritoneum 49 4 <1/3 low yes *KIT*  I (43.4)  bowel (M) exon 11  **G58** 64 72 M small liver 38 100 ≥1/3 high yes *KIT*  I (33.9)  bowel (M) exon 11  **G59** 58 48 F small bone 29 56 ≥1/3 high yes *KIT*  I (62.3),  bowel (M) exon 11 S (4.2),  R (9.4)  **G60** 65 69 M small peritoneum 57 27 ≥1/3 high no *KIT*  I (83),  bowel (M) exon 11 S (33.3)  **G63** 66 66 M small peritoneum 40 148 ≥1/3 low yes *KIT*  I (11.1)  bowel (M) exon 11  **G64** 67 53 M small liver 44 105 ≥1/3 low no *KIT*  I (50.8)  bowel (M) exon 11  **G65** 68 78 F stomach stomach 60 3 <1/3 low no *KIT*  None  (P) exon 11  **G66** 69 50 M stomach stomach 33 6 <1/3 low yes *KIT* None  (P) exon 11  **G70** 70 55 F stomach stomach 36 3 <1/3 low no *KIT*  None  (P) exon 11  **G73** 71 60 M stomach stomach 56 3 <1/3 low NE *KIT*  I (11.2)  (P) exon 11  **G74** 72 54 F small small 29 0 <1/3 low no *KIT* None  bowel bowel (P) exon 9  **G76** 73 46 F stomach stomach 37 2 <1/3 high no *KIT*  None  (P) exon 11  **G77** 74 56 M others rectum 65 23 <1/3 high yes *KIT* None  (P) exon 11  **G78** 75 41 M small peritoneum 41 42 <1/3 high no WT I (10.5),  bowel (M) S (21.8)  **G79** 76 79 M stomach stomach 42 1 <1/3 low no *KIT*  None  (P) exon 17  **G80** 77 47 F stomach stomach 25 0 <1/3 low no NE None  (P)  **G81** 78 78 M stomach stomach 27 7 <1/3 low yes NE None  (P)  **G83** 79 57 F stomach stomach 35 2 <1/3 low no *PDGFR* None  (P) exon 18  **G84** 80 57 F stomach liver 16 3 <1/3 low NE *KIT*  I (48.3)  (M) exon 11  **G85** 81 41 F stomach stomach 38 11 <1/3 low no *KIT* None  (P) exon 11  **G86** 82 57 M stomach stomach 22 0 <1/3 low no WT None  (P)  **G88** 83 77 M stomach stomach 29 3 <1/3 high no NE None  (P)  **G90** 84 62 F stomach liver 40 1 <1/3 high yes *PDGFR*  I (6.9),  (M) exon 18 S (2.2)  **G91** 85 60 F stomach stomach 25 49 <1/3 high no *KIT*  I (65.5)  (P) exon 11  **G92** 86 40 M small small 184 8 <1/3 low yes *KIT*  I (40.6),  bowel bowel (P) exon 11 S (3.7)  **G95** 87 61 F stomach stomach 62 8 <1/3 low no *KIT* None  (P) exon11  **G96** 88 72 F small liver 47 52 <1/3 high yes *KIT* I (29.6),  bowel (M) exon 9 S (37.4)  **G99** 89 71 F others rectum 46 0 <1/3 high no *KIT*  I (8.1)  (P) exon 11  **G100** 90 54 F small liver 26 40 <1/3 high yes *KIT*  I (54.2),  bowel (M) exon 11 S (1.5)  **G101** 60 39 M small peritoneum 36 38 <1/3 high yes *KIT* I (36.8),  bowel (M) exon 9 S (27)  **G107** 91 46 F stomach stomach 22 0 <1/3 low yes WT None  (P)  **G108** 92 60 M stomach stomach 50 2 <1/3 low no *KIT* None  (P) exon 11  **G109** 65 69 M small peritoneum 114 48 ≥1/3 high yes *KIT*  I (88.5),  bowel (M) exon 11 S (33.3)  **G110** 93 38 M stomach stomach 24 13 <1/3 low no WT None  (P)  **G112** 94 68 F small liver 29 14 <1/3 high no *KIT*  I (1.6),  bowel (M) exon 9 S (23.4)  **G113** 95 54 F small liver 96 49 ≥1/3 high yes *KIT* I (41.9),  bowel (M) exon 11 S (6.6),  R (12.7)  **G115** 96 64 F stomach stomach 58 2 <1/3 low no *PDGFR* None  (P) exon 18  **G118** 97 54 M others rectum 55 61 ≥1/3 high yes *KIT* None  (P) exon 11  **G120** 98 70 F small liver 55 23 ≥1/3 high no *KIT*  I (29.6)  bowel (M) exon 11  **G121** 99 59 M stomach stomach 30 12 <1/3 low no *KIT*  None  (P) exon 11  **G122** 100 76 F small small 29 6 <1/3 low no *KIT* None  bowel bowel (P) exon 11  **G123** 101 28 F stomach peritoneum 100 1 <1/3 low no WT I (69.8),  (M) S (8,5)  **G125** 102 55 F stomach stomach 21 3 <1/3 low no NE None  (P)  **G126** 103 53 F stomach stomach 49 4 <1/3 high no *PDGFR* None  (P) exon 18  **G128** 104 53 M small peritoneum 26 13 <1/3 low no *KIT* I (45.8)  bowel (M) exon 9  **G129** 105 63 F small small 65 5 <1/3 low yes *KIT*  None  bowel bowel (P) exon 9  **G130** 106 41 F stomach stomach 49 6 <1/3 high no *KIT*  None  (P) exon 11  **G132** 107 57 F small liver 37 64 ≥1/3 low yes *KIT*  I (14.8)  bowel (M) exon 11  **G133** 108 43 F small peritoneum 12 27 <1/3 low no *KIT*  I (112.1)  bowel (M) exon 17  **G134**  109 53 M stomach stomach 34 4 <1/3 low no NE None  (P)  **G135** 110 73 M stomach stomach 262 93 ≥1/3 high yes *KIT* I (16.7)  (P) exon 11  **G136** 22 62 F stomach peritoneum 39 9 <1/3 high no *KIT*  I (58.2)  (M) exon 11  **G138**  111 62 F stomach stomach 20 6 <1/3 high no *KIT* None  (P) exon 11  **G139** 112 62 M others rectum 33 3 <1/3 low no *KIT*  None  (P) exon 11  **G140** 113 49 M stomach stomach 28 3 <1/3 low no *KIT*  None  (P) exon 11  **G142** 114 64 F small peritoneum 35 17 <1/3 high no *KIT*  I (80.3)  bowel (M) exon 11  **G143** 115 77 M stomach stomach 31 2 <1/3 low no NE None  (P)  **G144** 116 70 F stomach stomach 26 7 <1/3 high no NE None  (P)  **G145** 117 68 M stomach stomach 40 8 <1/3 low no WT None  (P)  **G146** 118 76 F small small 32 2 <1/3 low no NE None  bowel bowel (P)  **G147** 119 73 F stomach stomach 26 1 <1/3 low no *KIT*  None  (P) exon 11  **G148** 120 39 M small peritoneum 96 94 <1/3 high yes *KIT*  I (8),  bowel (M) exon 11 S (1.2)  **G149** 121 74 F stomach stomach 35 6 <1/3 low no *KIT*  None  (P) exon 17  **G150** 122 43 M small small 61 2 <1/3 low no *KIT*  None  bowel bowel (P) exon 9  **G151** 123 53 M small peritoneum 62 16 <1/3 low no *KIT* I (44.8)  bowel (M) exon 9  **G152** 124 59 M small peritoneum 85 20 <1/3 high yes *KIT*  I (113),  bowel (M) exon 9 S (6.8)  **G153** 125 71 M small small 80 40 <1/3 high no *KIT*  None  bowel bowel (P) exon 11  **G157** 126 36 M stomach stomach 30 18 <1/3 high no WT None  (P)  **G158** 127 66 F small peritoneum 126 15 <1/3 low yes WT I (3.1),  bowel (M) S (0.8)  **G160** 128 61 F stomach peritoneum 49 50 <1/3 high yes *KIT* I (58.2),  (M) exon 11 S (27.4)  **G161** 129 57 F stomach stomach 46 2 <1/3 high no NE None  (P)  **G163** 130 52 M small peritoneum 48 55 <1/3 high yes *KIT* I (122.2)  bowel (M) exon 9  **G166** 131 59 M stomach stomach 20 1 <1/3 high no *PDGFR* None  (P) exon 18  **G167** 132 62 M stomach stomach 25 2 <1/3 low no *PDGFR*  None  (P) exon 18  **G168** 133 73 M stomach stomach 31 7 <1/3 low no *KIT*  None  (P) exon 11  **G170** 134 55 M stomach stomach 28 8 <1/3 low yes *KIT*  None  (P) exon 11  **G173** 135 51 F stomach stomach 76 3 <1/3 low yes WT None  (P)  **G174** 136 78 M others colon 50 11 <1/3 high yes *KIT* None  (P) exon 11  **G175** 137 42 M stomach stomach 31 7 <1/3 low yes WT None  (P)  **G177** 138 74 F stomach stomach 67 1 ≥ 1/3 low no *PDGFR*  None  (P) exon 18  **G178** 139 72 F stomach stomach 28 11 <1/3 high no *KIT*  None  (P) exon 11  **G179** 140 63 F small liver 37 24 <1/3 high yes *KIT* I (62.5),  bowel (M) exon 11 S (30.7)  **G181** 141 51 M stomach stomach 26 1 <1/3 low no *KIT*  None  (P) exon 11  **G183** 142 58 F stomach peritoneum 101 5 <1/3 high yes *KIT*  I (20.4),  (M) exon 11 S (27.7),  R (23.4)  **G184** 143 63 F small peritoneum 39 45 <1/3 high no *KIT*  I (82.1)  bowel (M) exon 9  **G185** 144 68 F small liver 21 4 <1/3 high no *KIT* I (11.2)  bowel (M) exon 11  **G186** 145 54 F small liver 185 5 <1/3 low yes WT I (34.5)  bowel (M)  **G188** 146 68 M stomach stomach 24 9 <1/3 high no KIT None  (P) exon 11  **G189** 147 68 M stomach stomach 31 2 <1/3 low no *KIT*  None  (P) exon 11  **G190** 148 88 F small small 33 4 <1/3 low no NE None  bowel bowel (P)  **G193** 149 61 M small peritoneum 62 54 ≥1/3 high yes *KIT*  I (29.7)  bowel (M) exon 11  **G194** 65 70 M small peritoneum 81 49 ≥1/3 high yes *KIT*  I (93.4),  bowel (M) exon 11 S (33.3)  **G199** 150 71 F small peritoneum 81 96 ≥1/3 high yes *KIT*  I (53.1)  bowel (M) exon 9  **G205** 151 59 M stomach peritoneum 40 46 ≥1/3 high no *KIT* I (14.4)  (M) exon 11  **G219** 152 74 F stomach liver 19 40 ≥1/3 low yes *KIT* I (41.3)  (M) exon 11  **G227** 153 64 F small liver 127 0 <1/3 low no *KIT*  I (28.3),  bowel (M) exon 11 S (3.2)  **G231** 154 59 M small small 65 15 <1/3 low no *KIT*  I (9.9)  bowel bowel (P) exon 9  **G233** 155 41 F small liver 89 1 <1/3 high yes *KIT* I (16.2)  bowel (M) exon 11  **G238** 156 67 M small liver 28 72 <1/3 high yes *KIT*  I (59.1),  bowel (M) exon 9 S (14.3)  **G241** 157 73 M stomach peritoneum 103 1 ≥1/3 low yes *KIT* I (20.9)  (M) exon 11  **G243** 158 54 F small peritoneum 40 40 <1/3 low yes *KIT*  I (64.1)  bowel (M) exon 11  **G244** 159 69 M small liver 68 54 ≥1/3 high yes *KIT*  I (36.4)  bowel (M) exon 11  **G245** 160 68 F small peritoneum 28 32 <1/3 high yes *KIT*  I (80.9),  bowel (M) exon 11 S (0.9)  **G248** 161 50 M small peritoneum 49 68 <1/3 high yes *KIT* I (119.4)  bowel (M) exon 11  **G249** 162 37 F stomach stomach 55 1 <1/3 low no *KIT*  I (12.9)  (P) exon 11  **G251** 163 48 F small liver 130 6 ≥1/3 low no *KIT* I (7.1)  bowel (M) exon 9  **G252** 164 73 M small peritoneum 34 12 ≥1/3 high yes *KIT*  I (130.3)  bowel (M) exon 11  **G253** 165 66 M small peritoneum 95 74 <1/3 high yes *KIT*  I (19.3)  bowel (M) exon 11  **G254** 166 68 F small peritoneum 126 0 <1/3 high yes *KIT*  I (6.4)  bowel (M) exon 11  **G256** 7 64 M small peritoneum 57 92 ≥1/3 high yes *KIT*  I (91.5),  bowel (M) exon 11 S (21.8),  R (14.2)  **G259** 167 60 F small liver 39 32 ≥1/3 high yes *KIT*  I (31.8)  bowel (M) exon 11  **G263** 168 59 F small small 110 2 <1/3 low yes WT None  bowel bowel (P)  **G265** 169 56 M small small 110 71 ≥1/3 high no *KIT* None  bowel bowel (P) exon 11  **G268**  170 69 M small peritoneum 50 208 ≥1/3 high yes *KIT*  I (37.9)  bowel (M) exon 9  **G269** 171 36 F stomach stomach 34 22 ≥1/3 low no WT None  (P)  **G270** 172 52 M small peritoneum 62 15 <1/3 low no *KIT* I (9.9),  bowel (M) exon 11 S (55.2)  **G275** 173 41 F stomach stomach 85 16 <1/3 high no *KIT*  None  (P) exon 11  **G277** 174 65 M stomach peritoneum 29 48 ≥1/3 high no *KIT* I (26)  (M) exon 11  **G283** 175 49 M others rectum 32 135 <1/3 high no *KIT*  I (19.1)  (P) exon 11  **G286** 176 71 F small liver 62 1 <1/3 high yes *KIT*  I (16.9)  bowel (M) exon 9  **G290**  107 59 F small peritoneum 37 0 <1/3 low no *KIT*  I (43.5)  bowel (M) exon 9 |

^a^M, metastasis; P, primary tumor

^b^I, imatinib; S, sunitinib; R, regorafenib; So, sorafenib

WT, wild type
