# Supplementary material for: Establishment of patient-derived xenografts from patients with gastrointestinal stromal tumors: analysis of clinicopathological characteristics related to engraftment success
Source: Sci Rep. 2020 May 14;10:7996. doi: 10.1038/s41598-020-64552-w (PMC7224375; doi:10.1038/s41598-020-64552-w)
Supplement: Supplementary file 2 — Supplementary information2. [file 41598_2020_64552_MOESM2_ESM.docx]

**Establishment of patient-derived xenografts from patients with gastrointestinal stromal tumors: analysis of clinicopathological characteristics related to engraftment success**

Young-Soon Na^1†^, Min-Hee Ryu^2†^, Young Soo Park^3^, Chae-Won Lee^1^, Ju-Kyung Lee^1^, Yangsoon Park^3^, Jung Min Park^1^, Jungeun Ma^2^, and Yoon-Koo Kang^2*^

^1^Asan Institute for Life Sciences, Asan Medical Center, University of Ulsan College of Medicine, Seoul, Korea

^2^Department of Oncology, Asan Medical Center, University of Ulsan College of Medicine, Seoul, Korea

^3^Department of Pathology, Asan Medical Center, University of Ulsan College of Medicine, Seoul, Korea

^†^Young-Soon Na and Min-Hee Ryu contributed equally as first authors to this work.

**Running title**: Clinicopathological factors related to engraftment success

**Keywords:** Gastrointestinal stromal tumor, patient-derived xenograft, clinicopathological characteristics, success factor

***Correspondence to:** Yoon-Koo Kang, MD, PhD, Department of Oncology, Asan Medical Center, University of Ulsan College of Medicine, 88, Olympic-ro 43-gil, Songpa-gu, Seoul 05505, Korea

Tel.: 82-2-3010-3230; Fax: 82-2-3010-8046; E-mail:[ykkang@amc.seoul.kr](mailto:ykkang@amc.seoul.kr)

Supplementary Table S2. The success rate according to the number of three factors related to success

| Number of ^a^factors N Success Success (%) 95% CI  related to success |
| --- |
| 0 44 0 0.0 0.0% 8.0%  1 50 2 4.0 1.1% 13.5%  2 56 10 17.9 10.0% 29.8%  3 35 19 54.3 38.2% 69.5% |
| Number of ^b^factors N Success Success (%) 95% CI  related to success |
| 0 59 0 0.0 0.0% 6.1%  1 61 6 9.8 4.6% 19.8%  2 49 15 30.6 19.5% 44.5%  3 16 10 62.5 38.6% 81.5% |

^a^factors related to success: Ki-67≥1/3, after TKI treatment, and largest tumor size >50

^b^factors related to success: Ki-67≥1/3, after TKI treatment, and largest tumor size >100

95% CI calculated by the Wilson score method
